# Conservation Genomics of the Declining North American Bumblebee *Bombus terricola* Reveals Inbreeding and Selection on Immune Genes

**DOI:** 10.3389/fgene.2018.00316

**Published:** 2018-08-10

**Authors:** Clement F. Kent, Alivia Dey, Harshilkumar Patel, Nadejda Tsvetkov, Tanushree Tiwari, Victoria J. MacPhail, Yann Gobeil, Brock A. Harpur, James Gurtowski, Michael C. Schatz, Sheila R. Colla, Amro Zayed

**Affiliations:** ^1^Department of Biology, York University, Toronto, ON, Canada; ^2^Wildlife Preservation Canada, Guelp, ON, Canada; ^3^Faculty of Environmental Studies, York University, Toronto, ON, Canada; ^4^FaunENord, Chibougamau, QC, Canada; ^5^Donnelly Centre, University of Toronto, Toronto, ON, Canada; ^6^Department of Molecular Genetics, University of Toronto, Toronto, ON, Canada; ^7^Simons Center for Quantitative Biology, Cold Spring Harbor Laboratory, Cold Spring Harbor, NY, United States; ^8^Departments of Computer Science and Biology, Johns Hopkins University, Baltimore, MD, United States

**Keywords:** *Bombus*, bumblebee, conservation, inbreeding, pathogen, genomics, population genetics

## Abstract

The yellow-banded bumblebee *Bombus terricola* was common in North America but has recently declined and is now on the IUCN Red List of threatened species. The causes of *B. terricola’*s decline are not well understood. Our objectives were to create a partial genome and then use this to estimate population data of conservation interest, and to determine whether genes showing signs of recent selection suggest a specific cause of decline. First, we generated a draft partial genome (contig set) for *B. terricola*, sequenced using Pacific Biosciences RS II at an average depth of 35×. Second, we sequenced the individual genomes of 22 bumblebee gynes from Ontario and Quebec using Illumina HiSeq 2500, each at an average depth of 20×, which were used to improve the PacBio genome calls and for population genetic analyses. The latter revealed that several samples had long runs of homozygosity, and individuals had high inbreeding coefficient F, consistent with low effective population size. Our data suggest that *B. terricola*’s effective population size has decreased orders of magnitude from pre-Holocene levels. We carried out tests of selection to identify genes that may have played a role in ameliorating environmental stressors underlying *B. terricola’*s decline. Several immune-related genes have signatures of recent positive selection, which is consistent with the pathogen-spillover hypothesis for *B. terricola*’s decline. The new *B. terricola* contig set can help solve the mystery of bumblebee decline by enabling functional genomics research to directly assess the health of pollinators and identify the stressors causing declines.

## Introduction

Pollinators are responsible for or augment the reproduction of 80–95% of all flowering plants (Ollerton et al., 2011), including many agricultural crops (Klein et al., 2007). Bumblebees (*Bombus* spp.) are important pollinators of many plants, particularly in North America and Europe (Kearns et al., 1998; Berenbaum et al., 2007). Their ability to ‘buzz’ pollinate (Plowright and Laverty, 1987; Dogterom et al., 1998) makes them more suitable than honey bees for pollination of certain plants. However, many species of bumblebees have experienced recent declines (e.g., Williams et al., 2009, 2014; Williams and Osborne, 2009; Cameron et al., 2011). In North America, members of the subgenus *Bombus sensu stricto* seem to be particularly vulnerable (Grixti et al., 2009; Cameron et al., 2011; Colla et al., 2012; Hatfield et al., 2015). These species share certain ecological characteristics, including having a short tongue length and early spring emergence (Colla, 2016). Several general hypotheses have been proposed to explain bumblebee declines. These include habitat loss, climate change and pathogen spillover – the introduction of novel pathogens from commercial honeybees and bumblebee colonies to native colonies (Grixti et al., 2009; Szabo et al., 2012; Kerr et al., 2015). The specific causes underlying the decline of any given species or population of bumblebees often remain elusive.

The rapid decline of *Bombus terricola* Kirby 1837, illustrates the difficulties with diagnosing bumblebee declines. *B. terricola* is a native species in North America inhabiting much of Canada and parts of the United States, from the Eastern Temperate and Boreal forest regions, south along higher elevations of the Appalachians, west through North Dakota and the Canadian Great Plains, to the Tundra and Taiga of Canada and the Mountain West (Williams et al., 2009; Williams et al., 2014). It nests underground and is often found close to or within wooded areas and wetlands (Williams et al., 2009, 2014). This species has been noted to have significantly declined both in relative abundance and distribution throughout its native range and is designated as ‘of conservation concern’ in Canada (Colla and Packer, 2008; Cameron et al., 2011; Hatfield et al., 2015) and is now on the IUCN Red List of threatened species (Hatfield et al., 2015; Spevak et al., 2015; Colla, 2016).

Conservation genomics – the use of high throughput genomics to study species targeted for conservation – is an emerging field that promises to transform conservation biology and pollinator conservation (Lozier and Zayed, 2016). Conservation genomics allows researchers to ask questions about species decline that were previously intractable using traditional (i.e., marker-based) population genetic methods. For example, researchers can directly quantify genetic diversity at phenotypically- and adaptively- relevant loci, and study how at-risk species are able to adapt to environmental stressors. Moreover, genomic tools and methods provide a framework for testing different hypotheses of species decline, by searching for transcriptomic or population genomic signals of response to stress (Lozier and Zayed, 2016).

Our objectives in this study were: to estimate the effective population size of *B. terricola* and related measures of possible inbreeding; to determine whether there are genes showing signs of recent selection; and, to find whether functional patterns in such genes help to distinguish between hypothesized causes of decline such as habitat loss, pesticides, climate change, and pathogen spillover.

Here we generated and analyzed several genomic datasets for the declining *B. terricola*, to aid in ongoing conservation efforts. Briefly, first we generated a draft partial genome or contig set, and associated gene predictions, using a long-read sequencing technology [Pacific Biosciences RS II (Rhoads and Au, 2015)]. Illumina HiSeq 2500 reads were used with Pilon (Walker et al., 2014) to iteratively refine the genome calls. *In silico* predictions of putative genes were then compared to the *B. terrestris* v1.0, and tools (Simão et al., 2015) were used to determine what proportion of a highly conserved arthropod genes set were in our gene set. We then used Illumina HiSeq 2500 to sequence the individual genomes of 22 further *B. terricola* samples from Ontario and Québec. Population genomics analyses showed evidence of recent declines in population size, and excess homozygosity across the genome indicative of inbreeding. Forensic-style analyses hinted at the role of pathogens as an important and recent selective pressure, which is consistent with the pathogen spillover hypothesis for *B. terricola*’s decline (Colla et al., 2006; Otterstatter and Thomson, 2008; Szabo et al., 2012; Graystock et al., 2016). Our sequencing costs and genomic analysis were suitable for conservation research on species without genomes. The genomic resources developed herein will facilitate future functional genomics research on healthy and declining *B. terricola* populations, to identify causes of their decline and to develop appropriate conservation management strategies for species recovery.

## Materials and Methods

### Sampling

The *B. terricola* specimens studied herein were sampled, in the provinces of Ontario and Québec, with a subset of sampled females used for sequencing as described below.

Surveys for *B. terricola* gynes occurred at over 30 sites within its historical range across Southern Ontario in April and May 2014; *B. terricola* gynes from 10 sites (**Supplementary Table S1**) were kept in the lab for behavioral study and prepared for sequencing if they died or at end of study period. Gynes were frozen in a household freezer as soon as they were found dead. In late July 2014, the frozen bees were transferred to a -80°C freezer at York University; 13 were used for genome sequencing and analysis.

Similarly, we also carried out surveys in northern Quebec near the towns of Chapais and Chibougamau in late May and June 2014. Twelve *B. terricola* gynes were sampled from nine sites (**Supplementary Table S1**). Gynes were monitored until natural death. While gynes were usually transferred to a -20°C freezer within 24–48 h of dying, some were left in their boxes for several days prior to freezing. Samples were packed in 95% ethanol and shipped to York University in January 2015; 9 were used for sequencing.

Our objective in this study was to limit the impact of our collections on the natural population. The number of gynes produced per colony of *B. terricola* is quite variable, which is particularly obvious in studies of captive bumblebees, with an average of 11 gynes produced per colony (Plowright, 1966; Plowright and Jay, 1968; Owen et al., 1980). Although any removal of gynes from an area carries the risk of reducing the overall population, the limited numbers collected in this study are unlikely to impact the population in a significant way.

**FIGURE 1:**
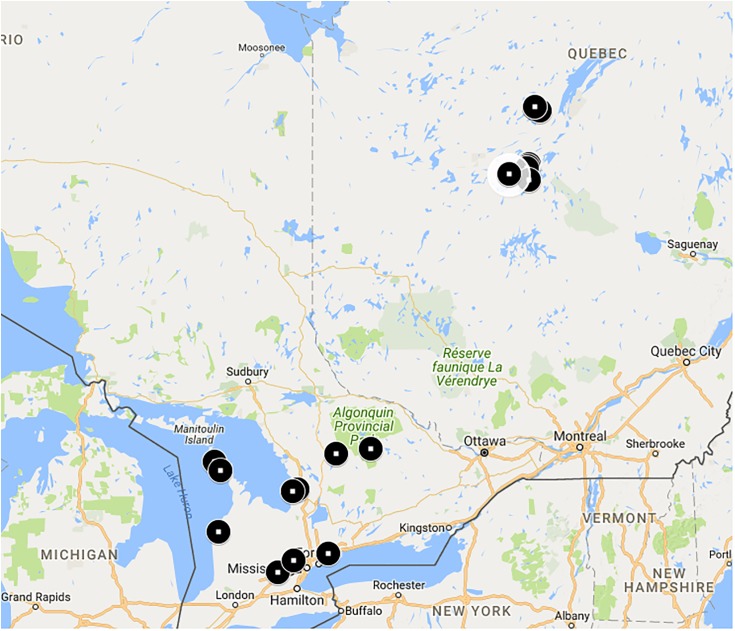
*Bombus terricola* collection sites. See **Supplementary Table S1** for details.

Our goal was to collect bees over a range of natural habitats and latitudes. Bees were collected from a number of sites (**Figure 1**) in Ontario and Québec, Canada (**Supplementary Table S1**). On average, Québec sites were approximately 500 km north of Ontario sites and were contained in an estimated area of 12,000 km^2^. Ontario sites were contained in an estimated area of 77,000 km^2^. Ontario sites are in the Eastern Great Lakes Lowland forests ecoregion while Québec sites are in the Eastern Canadian Forest-Boreal transition ecoregion (Dinerstein et al., 2017).

### DNA Extraction for the Reference Contig Assembly

High quality, high molecular weight genomic DNA was extracted from one *B. terricola* gyne using Qiagen Genomic-tip protocol and Qiagen Blood and Cell Culture DNA mini kit (Qiagen, Hilden, Germany) using manufacturer’s protocol with modifications in the lysis step. Specifically, 20 mg of bee tissue (comprising thorax and legs) was divided into two parts and lysis was carried out in two tubes of ∼10 mg tissue as starting material. For each part, the tissue was ground using liquid nitrogen, 350 μl CTAB extraction buffer (0.1 M Tris-HCl at ph8.0, 1.4 M NaCl, 0.02 M EDTA, 2% Cetyltrimethyl ammonium bromide) and 2 μl RNAse A added, and incubated at 37°C for 30 min. To this 20 μl Proteinase K and 1 ml Buffer G2 (supplied in the kit) was added and incubated overnight at 50°C. Next morning, the lysates from two tubes were vortexed and added to one Genomic-tip column. The protocol from here onwards was followed as specified in the kit’s manual. The size of the genomic DNA was evaluated on a pulse-field gel electrophoresis. The DNA was further quantified and checked for purity using Nanodrop. The high quality DNA isolated from this *B. terricola* gyne was sent to Génome Québec Innovation Centre (Montreal, Canada) for generating libraries and sequencing using the Pacific BioSciences RS II long read technology (Pacific Biosciences, Menlo Park, CA, United States). A total of 15 SMRT cells were used for this one sample to generate ∼35× coverage.

### DNA Extraction for Population Genomics

For the 22 samples of *B. terricola* from Ontario and Quebec, DNA was extracted from bee thoraces using Omega Bio-tek Magbind^®^ Blood DNA HDQ 96 kit (Omega Bio-tek Inc., Norcross, GA, United States) using manufacturer’s protocol. DNA quality was checked and quantified by standard agarose gel electrophoresis and Nanodrop. Subsequently DNA samples from these bees were sent to The Centre for Applied Genomics, Toronto, Canada for library preparation (using the Illumina TruSeq DNA PCR-Free kit) and sequencing by Illumina HiSeq 2500 at an average coverage of 20×.

### Reference Genome Assembly

The *B. terricola* contig set was assembled from the PacBio sequencing data of a single gyne using a self-correction and assembly approach derived from the PBcR algorithm (Koren et al., 2013). Briefly, all raw PacBio reads greater than 5 kbp long (max read length: 53,848 bp), representing approximately 35× fold coverage of the genome, were aligned to each other using the DAligner algorithm (Myers, 2014) using the parameters “–l2000 –M35.” After aligning the reads, the raw reads were error corrected by computing the consensus of how the reads aligned to a given read using the Dazcon algorithm with the parameters: “-xo -l 1000 -j 5 -c 4 -t 4 -m 450.” We used Daligner and Dazcon instead of the default PBcR components to accommodate the larger genome size as recommended by PacBio. The source code for these modules is available from the Pacific Biosciences Github repository: https://github.com/PacificBiosciences/pbdagcon.git, commit id: c2776be553e0408f77f1389e5ecddbc8a12fd619.

After error correction, the reads were assembled using the Celera Assembler 8.3rc2, which has been optimized for long read assembly (Koren et al., 2013). The Celera Assembler uses a string graph approach (Myers, 2005), where the long reads are overlapped to each other to form the initial graph, after which the graph was transformed and simplified to form the final contigs using the bogart unitigging algorithm (Koren et al., 2013). We used revision 4650 of the source code available from sourceforge here: svn: //svn.code.sf.net/p/wgs-assembler/svn/trunk/src. Based on the read length and coverage available after error correction, we used the following parameters for the assembly: frgMinLen = 3000, ovlMinLen = 200, ovlMerSize = 21, ovlErrorRate = 0.03, utgGraphErrorRate = 0.03, utgGenomeSize = 250000000, unitigger = bogart.

**FIGURE 2:**
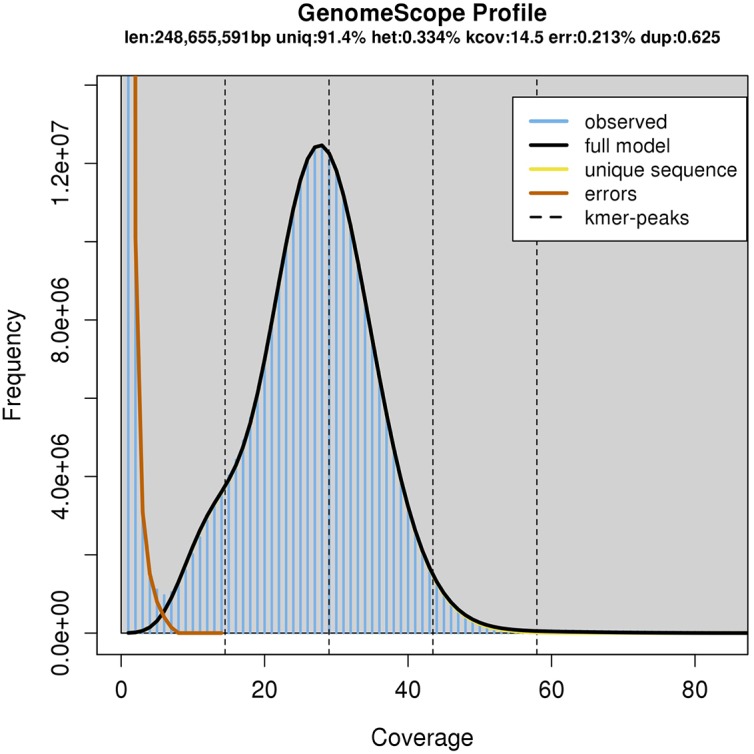
GenomeScope Analysis of the 23-mers in the Illumina sequencing data.

The resulting assembly consisted of 26,131 contigs ranging from 7,676 bp to 2,171,155 bp long. The NG50 size, meaning that half of the genome was assembled into contigs this size or larger, was 359 kbp assuming a genome size of 250 Mbp. However, the total span of the assembly was 566 Mbp, most likely because of the heterozygosity present in the sequencing data lead to a partially phased assembly (Chin et al., 2016). Indeed, using the GenomeScope algorithm (Vurture et al., 2017) with the available Illumina sequencing data, we estimate the genome size to be 248 Mbp (**Figure 2**).

We further improved the PacBio assembly based on Illumina sequencing derived from the same gyne (B28). Illumina reads from the same gyne were aligned to the PacBio-based assembly using NextGenMap (NGM) (Sedlazeck et al., 2013). Coverage levels for each contig were calculated; mean coverage was bimodal. A subset of contigs with coverage above a cutoff was chosen, and B28 Illumina reads were realigned to this high coverage subset. The resulting bam files and the subset of high coverage contigs were used to improve genome calls using Pilon in its diploid genome mode (Walker et al., 2014). B28 Illumina reads were realigned to the Pilon-revised genome and Pilon was called iteratively until relatively few changes were made in the second last iteration. In the final iteration the bam file produced by NGM was realigned using the GATK realignment pipeline (Auwera et al., 2013) before running Pilon. The Pilon-revised genome from the last iteration is version 1.0 of the contig set.

### Gene Prediction

Once a reference set of contigs had been prepared, our next goal was to call genes. We applied Augustus’s gene prediction algorithm (Stanke and Waack, 2003; Keller et al., 2011), using the *B. terrestris* training set (Sadd and Barribeau, 2015) to predict genes in *B. terricola*’s version 1.0 contig set. BUSCO (Simão et al., 2015) with the Arthropoda data set and with the *B. terrestris* training set (Sadd and Barribeau, 2015) was used to assess the completeness of contig assembly against a set of 2,675 conserved arthropod genes. Results from reciprocal Blastp best match (BBM) to *B. terrestris, B. impatiens* (Sadd and Barribeau, 2015), and *Apis mellifera* Official Gene Sets 1.0 (Weinstock et al., 2006) and 3.2 (Elsik et al., 2014) as well as additional genes found via proteomic analysis (McAfee et al., 2015) were used to provide a preliminary annotation of genes.

### Variant Discovery

After determining a set of genes, the next phase in the analysis was to find variation in those genes for population genomic analyses. Nine females from Québec and 13 females from Ontario were sequenced with Illumina, for a total of 22 diploid genomes at average coverage of 20×. We used GATK version 3 (McKenna et al., 2010) to call variants. All elements of the GATK best practices pipeline (Auwera et al., 2013) not requiring pre-existing validated SNP sets were used. We used GATK Haplotype Caller followed by GenotypeGVCF to jointly call variants. Single Nucleotide Polymorphisms (SNPs) within 10 bp of indels were removed from the variant set.

### Population Genomic Analyses

We used Admixture 1.3.0 (Alexander et al., 2009) with a set of variants chosen every 10,000 SNPs to avoid linkage disequilibrium. Cross-validation error *Q*-values from runs with from 1 to 5 populations were collated. The *K*-value with smallest *Q* is taken to be the best population number.

Given a set of variants in and near genes, we next performed population genetic analyses to determine characteristics of these variants. We analyzed variants using VCFtools (Danecek et al., 2011) to measure the individual inbreeding coefficient F, long runs of homozygosity LROH, Tajima’s *D*, nucleotide diversity π within each region, and the fixation index *F*_ST_ between Québec and Ontario bees. Synonymous and non-synonymous sites in predicted genes were called by SnpEFF (Cingolani et al., 2012).

Several population genomic methods look at regions larger than a gene to detect signs of selection, of which we used two, Identity By Descent (IBD) (Browning and Browning, 2013), and Haplotype differentiation. Chromosome regions Identical by Descent were identified after compensating for sequencing error using IBDseq (Browning and Browning, 2013). Using hapFLK 1.3 (Fariello et al., 2013) to compare haplotype differentiation with Québec and Ontario bees, we defined 702 contiguous regions with *p* < 0.01 at the center and for which all other SNPs in the region had *p* < 0.015 (hereafter called high-differentiation regions). We characterized genes in the lowest decile of *D* within these HapFlk regions as genes which vary between regions and which may be under directional selection (*D* < -1).

Strong recent selection produces linkage disequilibrium in the region under selection. We calculated linkage disequilibrium (*r*^2^) between SNPs using Plink 0.67 (Purcell et al., 2007) for non-inbred bees (*N* = 16) with minor allele frequency > 0.04, and corrected for sample size using Weir and Hill’s formula (Weir and Hill, 1980) and mutation rate using the method of Ohta and Kimura (1971) as modified in (Saura et al., 2015), using the value of alpha = 2 which includes mutation (Saura et al., 2015). We excluded inbred bees with *F* > 0.1 because inbreeding increases Linkage Disequilibrium (LD) without selection.

Effective population size *N*_e_ is an important measure in conservation genetics. *N*_e_ can be estimated in several ways (Saura et al., 2015; Waples et al., 2016). Recent *N*_e_ is best estimated using the temporal method (Saarinen et al., 2010), but our samples were collected during a single generation. Methods based on LD at various separations between SNPs give some insight into historical *N*_e_ – with closely separated SNPs providing information about the distant past – 10,000 to 1,000,000 years ago (Saura et al., 2015). We used NeEstimator v. 2.01 (Do et al., 2014) to estimate effective population size *N*_e_ in the past century. Both approaches are only accurate when LD is not relatively high, hence we excluded SNPs less than 10 bp apart. As noted in a recent study of using whole-genome SNP data for *N*_e_ estimation (Waples et al., 2016), using thousands of SNPs may not increase precision of estimates. We therefore prepared four independent, non-overlapping datasets: two SNP datasets (1st: 359 SNPs at least 10 Kbp apart, 2nd: 239 snps at least 15 Kbp apart) and two datasets with 110 and 429 widely spaced microsatellite sites. Microsats were included as there is an ongoing debate in the literature about whether SNP or microsat data is best used for estimating *N*_e_ in smaller population datasets (Morin et al., 2012), and we found NeEstimator produced finite upper CI estimates for the microsat data, but not the SNP data. NeEstimator was run with minimum Minor Allele Frequency (MAF) cutoffs of 0.05, 0.075, and 0.1. The values reported are for MAF = 0.075; the values found at MAF 0.05 and 0.10 are within the error bars for MAF 0.075. We used the LDNe method with the monogamous mating model, which is approximately correct for bumblebees (Duchateau et al., 1994). We also ran the heterozygote excess and molecular coancestry methods within NeEstimator to estimate very recent *N*_e_. The heterozygote excess method failed to converge and is not reported here. In the absence of a direct estimate of genome-wide recombination rate for *B. terricola*, we used the estimate of 4.4 cM/Mb found for *B. terrestris* (Stolle et al., 2011).

When a group of genes under selection share similar functions, this can hint at the type of selective pressures in action. Functional analysis of gene sets under selection was performed by mapping best matches of *B. terricola* genes to *Drosophila melanogaster* genes using blastp (reciprocal blast best match method) and OrthoDB v.9 (Kriventseva et al., 2015), which were then submitted to DAVID v. 6.7 (Huang et al., 2009). Genes under potential directional selection were determined by choosing genes overlapping windows in the lowest 5% of a weighted combination of *D* and log10(π), as shown in **Supplementary Figure S1** (red points). This selects for a large set of genes with Tajima’s *D* less than -2 and π less than the genome average, and a smaller set of genes with *D* < 0 and with π in the lowest 2% of the genome distribution. DAVID Functional Annotation Clustering was used with Biological Process, Level 5 annotations. A background list of all orthologous genes between *B. terricola* and *D. melanogaster* was used. Only clusters with an Enrichment Score greater than 2 are considered significant.

## Results

### Genome Size and Characteristics

The *B. terricola* contig set, constructed using Pacbio and Illumina sequencing, contains 238.9 million base pairs distributed over 1,448 contigs with an N50 of 341,136 bp (**Table 1**). The N50 contig size (341 Kb) of *B. terricola*’s genome is much larger than *B. terrestris* (76.0 Kb) and *B. impatiens* (57.1 Kb) recently sequenced using Roche 454 and Illumina technology (Sadd and Barribeau, 2015). Genome GC content (37.5%) is unimodal and not strongly skewed (**Supplementary Figure S2**), and similar to GC content of *B. impatiens* (37.9%) and *B. terrestris* (38.1%) (Sadd et al., 2015). We used an *in silico* algorithm Augustus (Stanke and Waack, 2003; Keller et al., 2011) to predict 15,100 putative genes in the *B. terricola* genome (see NCBI BioProject PRJNA399520 and linked genome files). We used local tblastn (Camacho et al., 2009) to compare the protein sequences called to the *B. terrestris* v1.0 genome. Median percent identity was 91.8% (1st quartile 83.0%, 3rd quartile 97.5%). We ran BUSCO (Simão et al., 2015) to determine how many of 2,675 highly conserved arthropod genomes were in our gene set; 94% of conserved genes were present, 1.5% were fragmented, and 4.3% were missing.

**Table 1:** Genome assembly statistics.

Reference genome coverage	35×	Population resequencing coverage	20×
Pacific BioScience RSII read size	5–54k	Illumina HiSeq v4 read len	2×150
Reference NG50 contig size	359k	Number of individuals	22
Min contig size	7.7k	Genome size	248M
Max contig size	2171k	GC content	37.5%
# contigs	26,131	Genes called	15,100


*The genome of Bombus terricola was assembled from Pacific Biosciences RS II reads for a single specimen, plus Illumina reads for the same specimen and 21 others. k, kbp; M, Mbp*.

### Population Genomics

#### Low Population Structure but Typical Levels of Genetic Diversity

Because our Ontario and Québec sampling sites were far apart and in largely different ecozones, we needed to determine whether they represented a single population. Bayesian analysis of population structure using Admixture (Alexander et al., 2009) revealed that *B. terricola* gynes from Québec and Ontario most likely belonged to the same population (*K* = 1) (**Supplementary Figure S3**). The average population differentiation as measured by Wright’s *F*_ST_ in 5 Kbp windows was also low at 0.008, so below we report whole-dataset statistics combining Québec and Ontario bees, unless noted otherwise. Nucleotide diversity as measured by π (Nei and Li, 1979) is 0.0024 (no minor allele frequency cutoff) to 0.0019 (using MAF = 0.09), similar to values found for π in *B. impatiens* (0.0024–0.0027) and *B. pensylvanicus* (0.0026–0.0029) (Lozier, 2014).

#### Several *B. terricola* Samples Show Signs of Inbreeding

Inbreeding is a major threat to bee populations because of the peculiarities of the Complementary Sex Determination (Csd) system (Zayed, 2004; Zayed et al., 2004; Zayed and Packer, 2005). Accordingly we calculated several statistics that measure inbreeding. The Québec population had 4/9 individuals (44%) with Wright’s inbreeding coefficient *F* (Wright, 1965) greater than 0.1, while the Ontario population had only 1/13 (7.7%). This difference was not significant by Fisher’s Exact test (odds ratio = 8.56, *p* = 0.116). Over the whole dataset, *F* had median 0.013, mean 0.036, 3rd quartile 0.077, and maximum 0.217.

The distribution of Long Runs of Homozygosity (LROH) is affected by inbreeding, and the inbreeding coefficient in individuals is highly correlated with their log-transformed total base pairs in LROH (Spearman’s *r* = 0.76, df = 20, *p* = 0.00005; **Supplementary Figure S4**). One inbred bee had two LROH segments each 1.3 Mbp in length.

#### The Effective Population Size for *B. terricola* Is Declining

**FIGURE 3:**
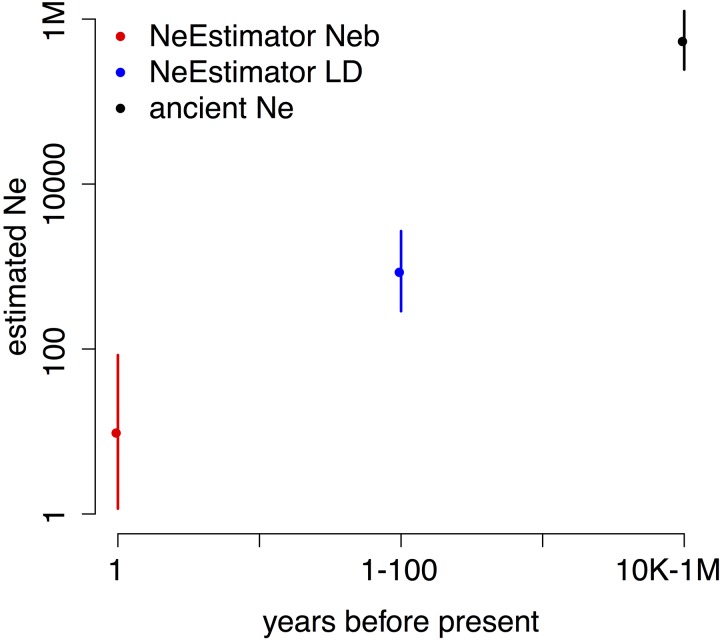
Estimated *N*_eb_ (breeding *N*_e_, red), recent *N*_e_ (blue), and ancient *N*_e_ (black) show orders of magnitude declines from ancient to recent to previous generation timescales. Note the logarithmic scale on both axes. Shown are means ± 2 SD; the error bars for ancient *N*_e_ lie within the point.

To estimate ancient *N*_e_, we used three methods based on LD between SNPs (Methods). Each estimates *N*_e_ at a different time in the past, giving the three time points shown in **Figure 3**. We ran each method on multiple datasets of SNPs (Methods) to provide the 2 standard error bars in the figure. The analysis shows a large decline in effective population size that likely occurred within the past 100 years, consistent with ecological evidence of decline for this species.

#### Loci With Signs of Selection Highlight Possible Adaptive Responses to Stressors

Stressors underlying *B. terricola’*s decline may have led to recent adaptive changes that can be revealed through population genomic analysis. We quantified four population genetic statistics: genetic diversity π, Tajima’s *D*, linkage disequilibrium (LD), and regions of Identity By Descent (IBD). Positive selection is expected to reduce π and *D* while increasing LD and IBD around selected loci. We first report the whole-genome functional classification of genes with low π and *D*, then provide additional evidence of positive selection on a targeted immune gene set using LD and IBD data.

Genes in the lowest portion 5% of the joint distribution of *D* and π (Methods) had 10 significantly enriched Functional Biological Process categories (**Supplementary Table S2**). Among these, cluster 2 contains GO term “MAPK cascade,” cluster 4 has “cellular macromolecule catabolic process,” and cluster 7 includes “regulation of innate immune response.” The latter cluster was intriguing given that pathogen spillover is one hypothesized cause of *B. terricola*’s decline (Colla et al., 2006; Otterstatter and Thomson, 2008; Sachman-Ruiz et al., 2015). Standing variation in genes giving resistance to pathogens may be quickly selected under such conditions (Przeworski et al., 2005; Reid et al., 2016). We thus restricted our search to a candidate gene set – those involved in immunity in honey bees (Ryabov et al., 2014), supplemented by homologs from *D. melanogaster* genes annotated as associated with Gene Ontology Biological Process term “Immune System Process,” GO: 0002376 (**Supplementary Table S3**).

**FIGURE 4:**
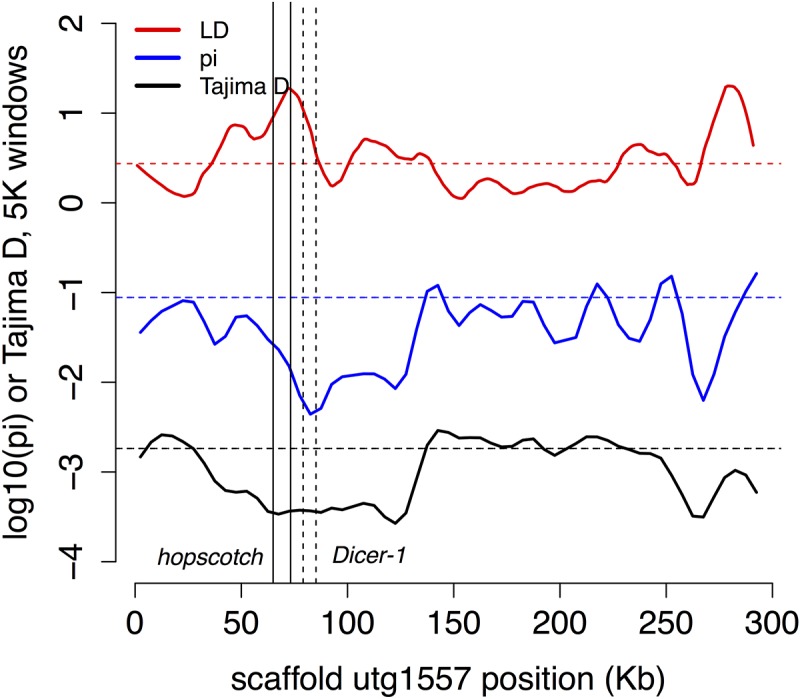
Possible selection on two immune genes. The region of high LD and low π and Tajima’s *D* contains the two immune genes Dicer-1 (solid lines) and hopscotch/JAK (dashed lines), which are also found in shared regions of IBD. In this and subsequent figures we plot Tajima’s *D* in 5 Kb windows (black), –log_10_(π) in 5 Kb windows (blue), and a 2 Kb window estimate of LD (red; as expected *r*^2^ at 20 bp; see Methods), with dashed horizontal lines in each color marking the local genome average for the measure.

Innate immune genes with two or more population genetic statistics consistent with directional selection are listed in **Table 2**. We found one region of shared IBD, high LD and low Tajima’s *D* and π contains the genes *hopscotch* and *Dicer-1* (**Figure 4**). This region contains above average LD, low π and Tajima’s *D*. The distribution of lengths of IBD regions is shown in **Supplementary Figure S5**.

**Table 2:** Immune-related genes with signs of selection.

*Drosophila melanogaster*	*Apis mellifera*	*Bombus terricola*	*Homo sapiens*	π	*D*	LD	IBD	HapFlk
*Rel*	GB40654	BT6895	NF-κB	^∗^	^∗^	^∗^		
*hop*	GB44594	BT7675	multiple	^∗^	^∗^	^∗^	^∗^	
*Dcr-1*	GB44595	BT7673	DICER1		^∗^	^∗^	^∗^	
*CG1667*	GB51714	BT3228	TMEM173	^∗^	^∗^	^∗^		
*CG4552*	GB46042	BT13432	TBC1D23	^∗^	^∗^	^∗^		
*smt3*	GB49167	BT3658	(SUMO)	^∗^	^∗^	^∗^		
*Ubr3*	GB49164	BT3659	UBR3	^∗^	^∗^	^∗^		
*Fhos*	GB43054	BT12525	FHOD1		^∗^	^∗^		^∗^
*scny*	GB47732	BT9755	USP36	^∗^	^∗^	^∗^		^∗^
*Ocrl*	GB40672	BT6892	OCRL	^∗^	^∗^			^∗^
*Lamp1*	GB49607	BT13031	LAMP1		^∗^			^∗^
–	GB41980	BT3224	AMBRA1	^∗^	^∗^			^∗^
*CG11652*	GB44355	BT9367	DPH1	^∗^	^∗^	^∗^		^∗^
*Atg14*	GB40935	BT2491	ATG14	^∗^	^∗^	^∗^	^∗^	
*senju*	GB46043	BT13433	multiple	^∗^	^∗^		^∗^	^∗^
*tefu*	GB50923	BT9874	ATM	^∗^	^∗^	^∗^		
*akirin*	GB51067	BT1008	AKIRIN1/2	^∗^	^∗^		^∗^	
*Tak1*	GB48187/8	BT8571	multiple	^∗^	^∗^		^∗^	
*E(bx)*	GB49172	BT3666	multiple	^∗^	^∗^			
*GstO2*	GB44803	BT12997	GSTO1/2	^∗^	^∗^			
*faf*	GB55472	BT12164	USP9X/Y	^∗^	^∗^			
*mor*	GB50017	BT1530	SMARCC1/2	^∗^	^∗^			
*POSH*	GB55569	BT13867	SH3RF1/3	^∗^	^∗^			
*IKK𝜀*	GB42451	BT11460	TBK1		^∗^			^∗^
*Uba2*	GB54228	BT8786	UBA2	^∗^	^∗^			
*hep*	GB51894	BT2561	multiple	^∗^	^∗^			


*Asterisk marks a population genetic measure which is in the direction consistent with positive selection. Genes with three or more of the five measures consistent with selection are top candidates. **Supplementary Table S4** gives details of mutations in these genes classified by SnpEff.*

**FIGURE 5:**
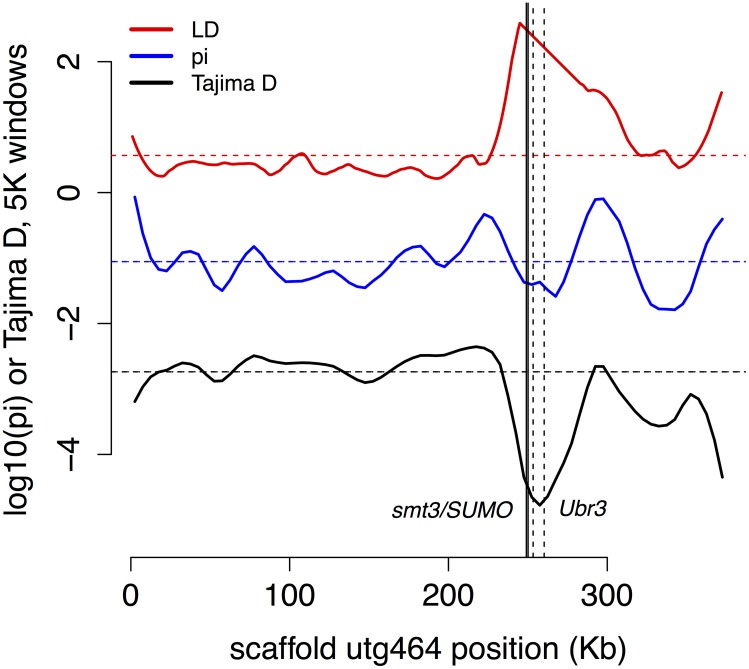
Partial sweep. Two genes (smt3/SUMO, solid lines, and Ubr3, dashed lines) involved in post-translational modification of immune genes show signs of recent selection.

We found a partial sweep (region of low genetic diversity π, low Tajima’s *D*, and high linkage disequilibrium) in the vicinity of two genes involved in post-translational modification, an important regulator of insect immune systems, that are adjacent to each other on scaffold 464 (**Figure 5**) for genes BT3658 (Amel GB49167, Dmel smt3, a.k.a. SUMO) and gene BT3659 (GB49164, Dmel Ubr3, e3 ubiquitin-protein ligase UBR3-like).

**FIGURE 6:**
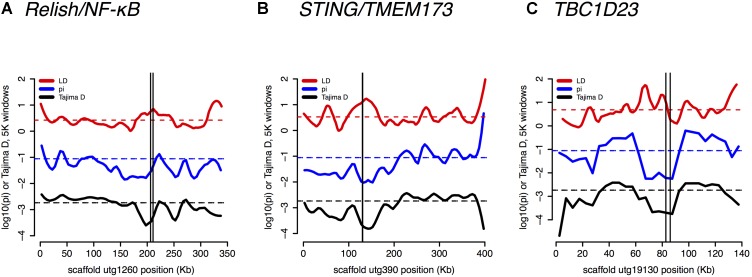
Linkage Disequilibrium (LD), π, and Tajima’s *D* are plotted for **(A)** Relish/NF-κB – a core immune signal transducer; **(B)** STING/TMEM173 – a transducer of viral RNA signal; and for **(C)** TBC1D23 – a negative regulator of Toll signaling.

Several core immune genes have the high LD and low π and Tajima’s D consistent with recent positive selection. Below, we plot these measures (**Figure 6**): for (a) Relish/NF-κB, (b) STING/TMEM173, and (c) TBC1D23.

#### Population Genomic Analysis of Local Adaptation

Some stressors, such as climate change, exposure to agrochemicals, and proximity to vegetable greenhouses that use commercial bumblebees for pollination, have a spatial component and are not expected to influence *B. terricola* across all of its native range. Climate change, for example, has different effects on bumblebee populations at the southern and northern ends of their ranges (Kerr et al., 2015), and our Québec sites are farther from greenhouses than Ontario sites. We thus queried our population genomic dataset to find genomic regions with signs of local adaptation – evidenced by extreme levels of haplotype differentiation (Albrechtsen et al., 2010) between Ontario and Québec. Using hapFLK 1.3 (Fariello et al., 2013) we defined 702 contiguous regions with *p* < 0.01 at the center and for which all other SNPs in the region had *p* < 0.015 (hereafter called high-differentiation regions). These high-differentiation regions covered 0.64% of the *B. terricola* genome. The median length of a region was 1098 bp (interquartile range 341-2379). Maximum *F*_st_ within these high-differentiation regions was in the upper 97.7% of the distribution of *F*_st_, and mean *F*_st_ over the regions was in the upper 96.2% of the distribution of *F*_st_. 259 genes (1.7% of all genes) overlapped these regions. Functional annotation of these genes produced 9 enrichment clusters with developmental, cell signaling, or neural functions. However, of the 24 immune genes in **Table 2** with at least two other signs of selection, 6 (25%) overlapped with HapFlk high differentiation regions. This odds ratio (25%/1.7%) is highly significant (χ^2^ = 75.5, df = 1, *p* = 3.7 × 10^-18^). Thus, immune genes with other signs of selection are significantly more likely than other genes to overlap regions of high differentiation between Ontario and Québec.

## Discussion

*Bombus terricola* was once common over much of Canada and the United States, but has been greatly reduced or extirpated throughout much of its historical range (Hatfield et al., 2015). We have assembled the draft *B. terricola* contig set and sequenced 22 bees from Ontario and Québec, Canada in an effort to develop resources and knowledge to help in the conservation management of this and other important pollinators.

The use of long-read sequencing technology allowed us to assemble a draft contig set for *B. terricola*, derived from a single gyne from Ontario. The genome size of *B. terricola* is similar to two other sequenced bumble bees (Sadd and Barribeau, 2015), but the PacBio assembly for the *B. terricola* genome is much improved over the older approaches [N50 contig size 341 Kb compared to *B. terrestris* (76.0 Kb) and *B. impatiens* (57.1 Kb)] (Sadd and Barribeau, 2015). We used *in silico* approaches to predict and annotate 15,100 genes in *B. terricola*, which is similar to the number of genes predicted in honey bees [*N* = 15,314 (Elsik et al., 2014)], and other bumblebees [11,669 (Sadd and Barribeau, 2015)]. *B. terricola*’s gene set included 94% of a set of highly conserved arthropod genes. While the quality of gene predictions for the *B. terricola* genome will surely benefit from RNA sequencing work under way, the current gene and contig set immediately enables researchers to carry out gene-centric evolutionary and conservation research.

In addition to generating genomic resources for *B. terricola*, we carried out a population genomic analysis to estimate classical conservation parameters (e.g., effective population size, prevalence of inbreeding and genetic differentiation) (Zayed, 2009). We found that, in the sampled areas, *B. terricola* is showing signs of inbreeding. This is of concern because complementary sex determination increases extinction risk of bees due to the diploid male vortex (Zayed and Packer, 2005). Increased probabilities of identity by descent within a population increase frequencies of diploid male production over those expected from random mating (Packer and Owen, 2001) because of the increased chances of a mating pair sharing a sex-determining allele in common. Inbred bee populations produce higher numbers of infertile diploid males instead of workers and gynes (Duchateau et al., 1994). At the mean *F*-value we measured, results from Packer and Owen (2001) suggest that up to 15% of gynes may be mating with males with a matching sex determination allele, resulting in a 50% loss of workers and probable failure or major size reduction of the resulting colonies (Duchateau et al., 1994; Darvill et al., 2006). At the highest values of *F* observed, up to 40% of gynes are mating with a male sharing a sex allele in common. Based on our analysis, we expect high levels of diploid male production in *B. terricola* populations – a prediction that can be validated by genotyping males collected during the early stages of the colony cycles – a time where only workers are typically produced.

Effective population size, *N*_e_, is an important parameter in conservation genetics, and conservation efforts strive to increase *N*_e_. Using LD at closely spaced variants, we were able to estimate the ancestral long-term harmonic mean *N*_e_ (**Figure 3**) in the period before the Holocene warming to be in the range of 400,000–600,000. Several methods which estimate recent *N*_e_ from recent times gave results orders of magnitude below pre-Holocene levels: around *N*_e_ = 950 for the LDNe method (last 100 generations) and *N*_eb_ = 10.2 for the molecular coancestry method (last generation) (Do et al., 2014). These dramatically smaller estimates suggest a recent major reduction in effective population size in *B. terricola.*

This potential recent drop in *N*_e_ must be considered in conjunction with other Quaternary-Holocene changes to explain the distribution of other population genetic statistics. For example, Tajima’s *D* normally shows a positive bias after a major population bottleneck (Tajima, 1989), but we observe mean *D* = -1.06. However, the current distribution of *B. terricola* is restricted to northern parts of the continent, suggesting that a bottleneck during the glaciations may have been followed by a rapid expansion during the post-glacial period. This would have produced a bias to negative values of *D* (Tajima, 1989; Wall and Przeworski, 2000). *D* changes on the timescale of 3*N*_e_ generations in haplo-diploid bees (Tajima, 1989; Wall and Przeworski, 2000) so our results may be due to a composite of post-glacial expansion and recent decline.

We used population genomic analysis to provide clues and insights into the environmental stressors that caused *B. terricola*’s decline. We hypothesized that environmental stressors leading to *B. terricola*’s decline may have imposed a selective pressure that, in turn, led to adaptive evolution at specific loci that ameliorated the negative effects of such stressors. As such, knowledge of the loci with signs of positive selection may hint at the causes of *B. terricola*’s decline. We note, however, that environmental stressors may not always lead to a quantifiable adaptive change in a genome; for example, there may have been no heritable differences in susceptibility to a stressor to fuel adaptive change within a population. Nevertheless, we think the approach is still valuable in highlighting genomic regions of adaptive significance to help generate and/or corroborate hypotheses for decline.

Ecological studies have highlighted stressors including habitat loss, climate change, pesticides, and pathogen spillover (Grixti et al., 2009; Szabo et al., 2012; Kerr et al., 2015) as likely causes for *B. terricola*’s decline. Gene ontology analysis highlighted innate immunity as an important biological process that is enriched among genes with recent signs of positive selection in *B. terricola*. These genes included regulators of immune signaling Relish/NF-κB, hopscotch/JAK, TBC1D23, smt3/SUMO, and Ubr3, anti-viral defense genes Dicer-1 and STING/TMEM173. Several genes found in regions of high differentiation between Québec and Ontario bees which also have low Tajima’s *D* have been associated with defense responses in fruit flies. It is possible that some pathogens differ between the two regions, as our Québec sampling sites are distant from greenhouse sites where introduced European pathogens were accidentally released.

We suggest that these signatures of selection may indicate that pathogen spillover is playing a role in *B. terricola* range reduction. We note, however, that positive selection on innate immune genes may not be unique to *B. terricola.* A recent study of several bee species found evidence of positive selection on two of the above mentioned genes: hopscotch/JAK, and Relish/NF-κB (Barribeau et al., 2015). Thus, some of our putative targets of recent selection may have also been under selection before the range decline.

## Conclusion

We show that assembly of a draft contig set and gene set combined with population genomics permits conclusions about a species at risk, at modest cost. Our results suggest that *B. terricola* populations in eastern Canada are experiencing inbreeding and declines in effective population size which may lead to colony losses via diploid male production. Signs of recent positive selection on a number of core and auxiliary immune genes suggest these bees may be experiencing novel pathogen pressures. Future areas of study of importance to conservation include completing a recombination map and chromosome assemblies to facilitate estimation of *N*_e_, direct sampling of pathogens in wild-caught bees, and population genetic sampling in northern areas more remote from greenhouse tomato and pepper operations. Moreover, transcriptomic studies of key tissues responsible for innate immune and detoxification across stable and declining *B. terricola* populations will be very helpful in ascertaining the stressors underlying declines of this species.

## Ethics Statement

Our study conforms to York University and Canadian guidelines on research involving lower animals.

## Data Accessibility

DNA data: NCBI Bioproject ID PRJNA399520 and linked genome, SRA entries.

## Author Contributions

VMP and YG collected samples. AD extracted DNA and performed initial diversity analyses with BAH. JG and MS assembled scaffolds from PacBio data. TT aligned Illumina samples and called SNPs. HP and NT assembled reference immune and detoxification gene sets and calculated population statistics on these genes. CK refined contigs, called gene sets, calculated genomic statistics, analyzed the data, and wrote the paper. VMP designed and supervised sampling plans with guidance by SC. AZ designed and supervised genomics work. SC, CK, and AZ edited the paper.

## Conflict of Interest Statement

The authors declare that the research was conducted in the absence of any commercial or financial relationships that could be construed as a potential conflict of interest.
