# Supplementary material for: Conservation Genomics of the Declining North American Bumblebee Bombus terricola Reveals Inbreeding and Selection on Immune Genes
Source: Front Genet. 2018 Aug 10;9:316. doi: 10.3389/fgene.2018.00316 (PMC6095975; doi:10.3389/fgene.2018.00316)
Supplement: Supplementary file 1 [file Table_1.DOCX]

**Supplemental Information for:**

Conservation genomics of a declining North American bumblebee,

*Bombus terricola,* reveal inbreeding and selection on immune genes.

Clement F. Kent, Alivia Dey, Harshilkumar Patel, Nadejda Tsvetkov, Tanushree Tiwari,

Victoria J. MacPhail, Yann Gobeil, Brock A. Harpur, James Gurtowski,

Michael C. Schatz, Sheila R. Colla, Amro Zayed

**Table of Contents:**

| **Supplementary Figure 1** | Page 2 |
| --- | --- |
| **Supplementary Figure 2** | Page 3 |
| **Supplementary Figure 3** | Page 4 |
| **Supplementary Figure 4** | Page 5 |
| **Supplementary Figure 5** | Page 6 |
| **Supplementary Table 1** | Page 7 |
| **Supplementary Table 2** | Page 8 |
| **Supplementary Table 3** | Page 8 |
| **Supplementary References** | Page 8 |

## Supplementary Figure 1

Values of Tajima’s D (horizontal axis) and nucleotide diversity π (log10 transformed, vertical axis) are shown genome wide. Regions shown in red are in the lower 2.5% of the joint distribution of D and pi. Genes in red regions were analysed for functional groups.


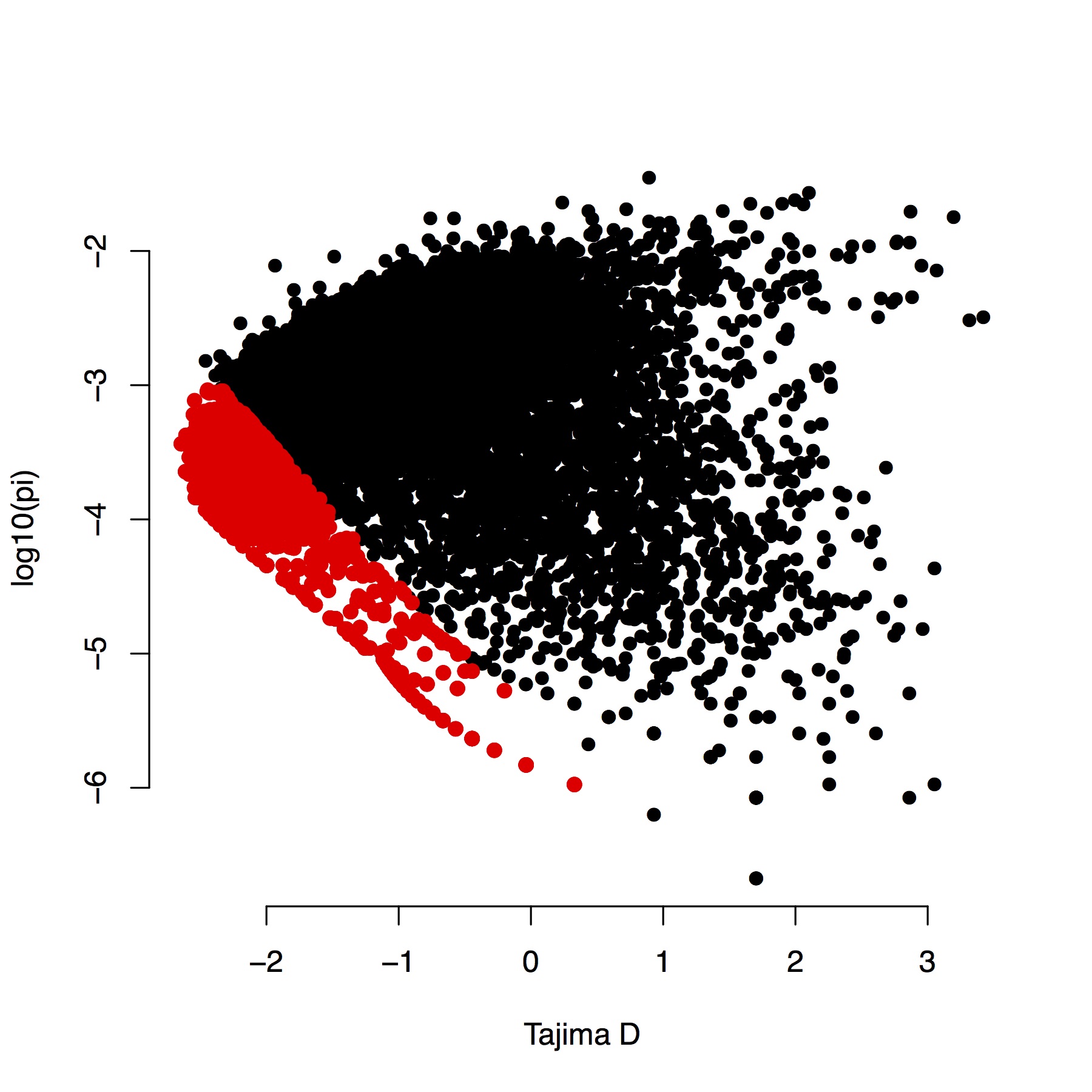


## Supplementary Figure 2

The distribution of GC content by contigs in the genome is unimodal.


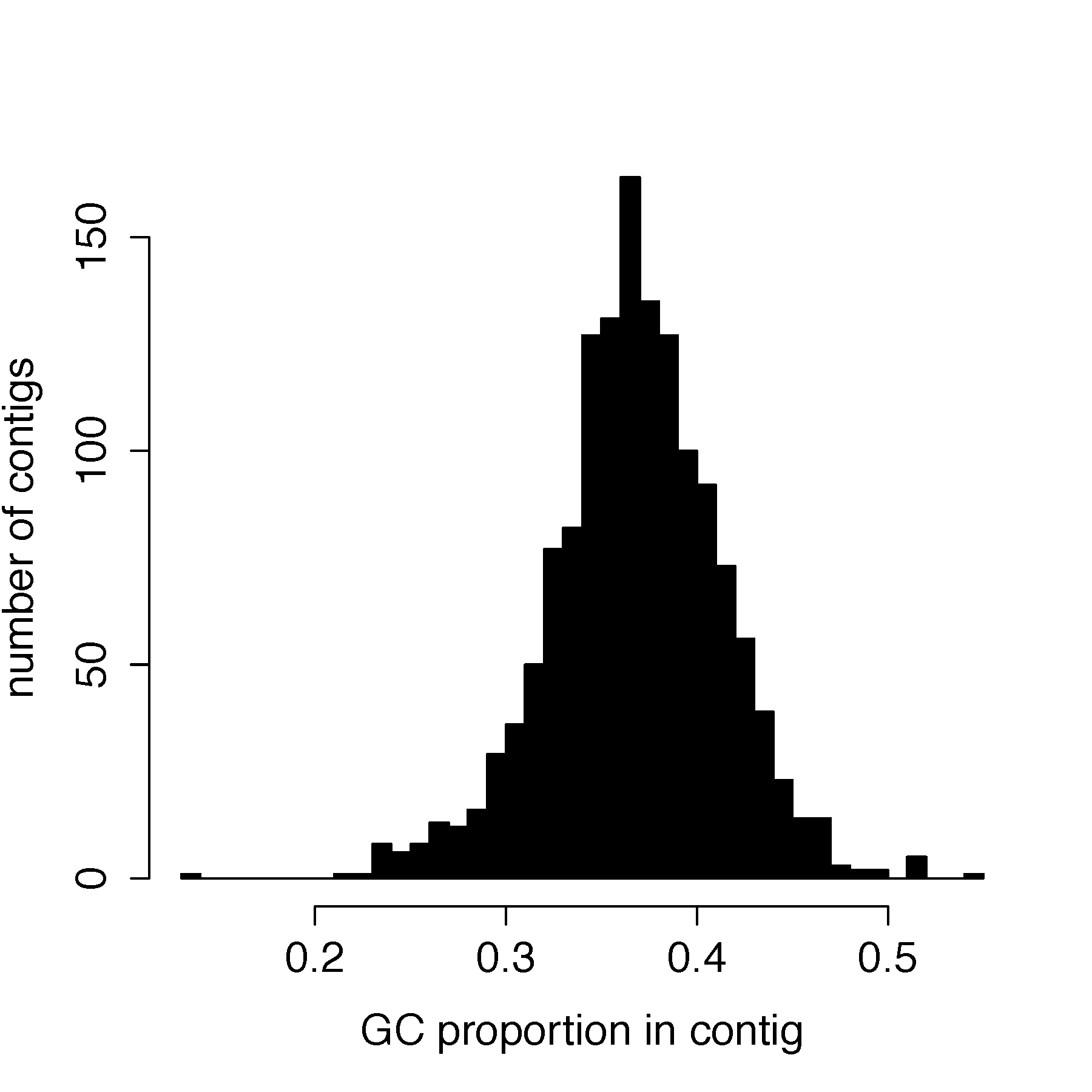


## Supplementary Figure 3

The plot shows the cross-validation error Q produced by the Admixture program for different numbers of populations. A single population has the lowest error Q.


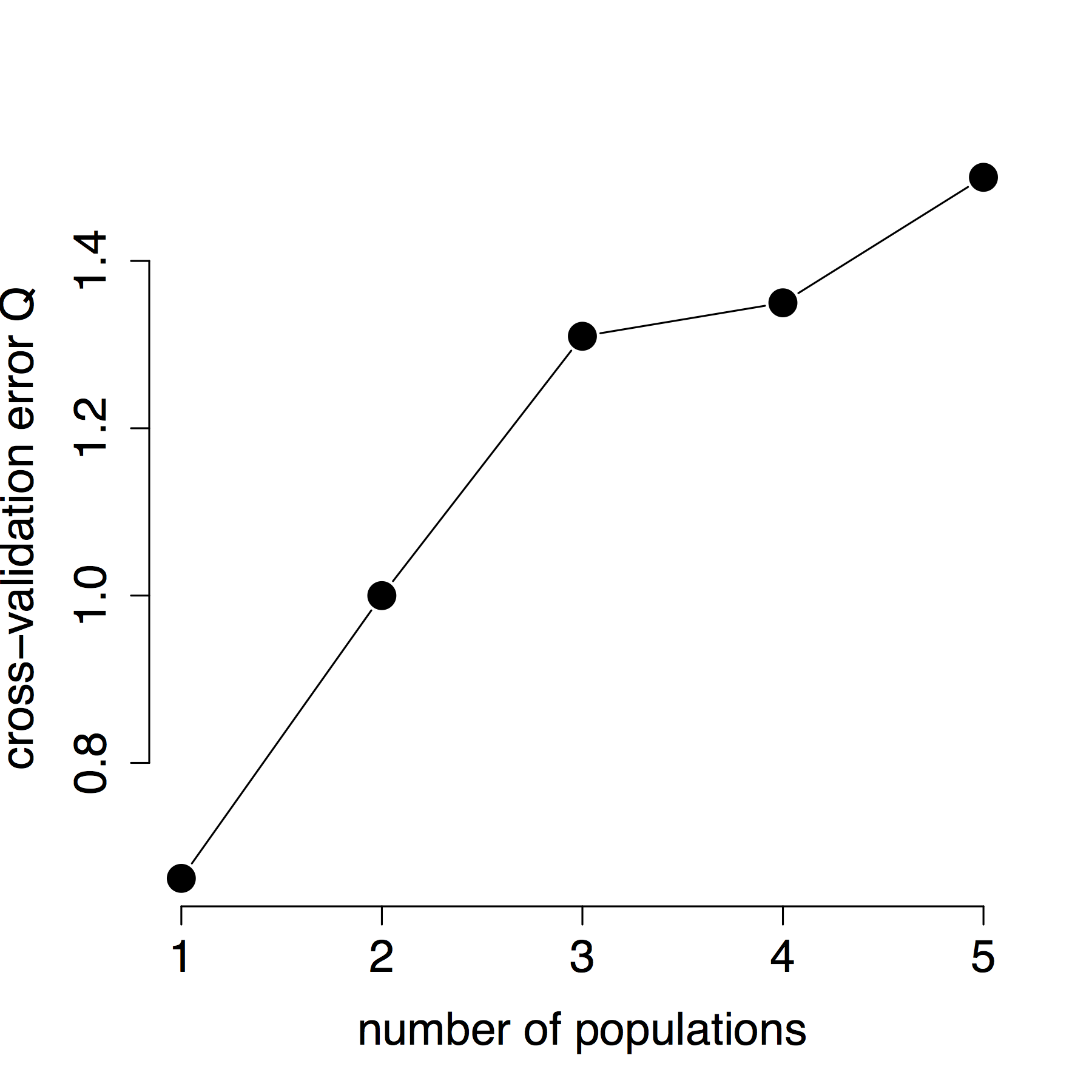


## Supplementary Figure 4

Inbreeding produces a high F statistic (horizontal axis) and total length per bee of Long Runs Of Homozygosity (vertical axis, Kb). Quebec bees are blue, Ontario bees black. There is a positive correlation (Spearman’s r = 0.76, df=20, p = 0.00005) between the two measures.


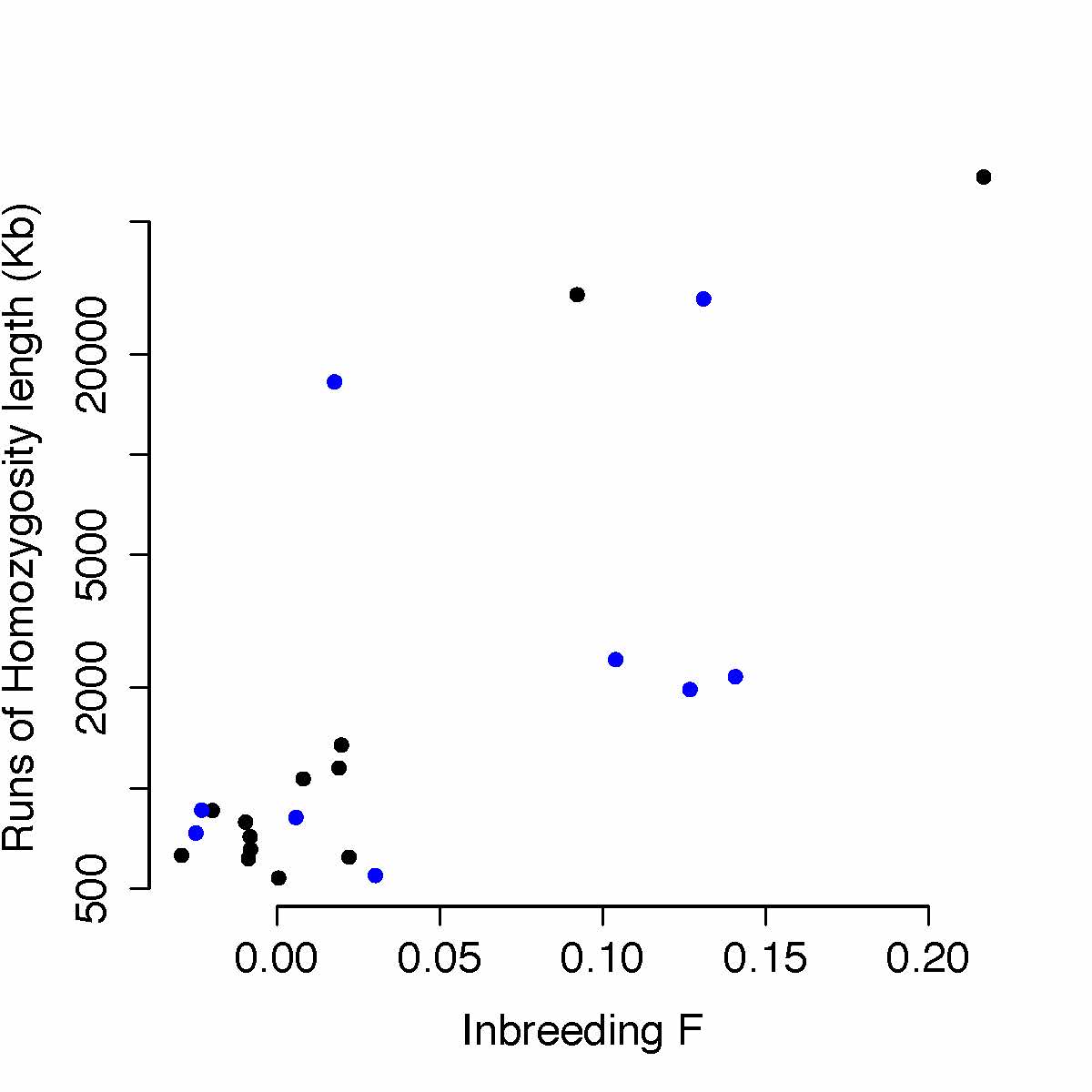


## Supplementary Figure 5.

Identity By Descent of tracts in the genome have lengths affected by time to coalescence, selection, and effective population size(Albrechtsen, Moltke, & Nielsen, 2010). The vertical axis plots the number of base pairs in the genome which are in IBD tracts shared between any two bees, in bins (on the horizontal axis) of the length of those tracts (each bin is 50Kb wide).


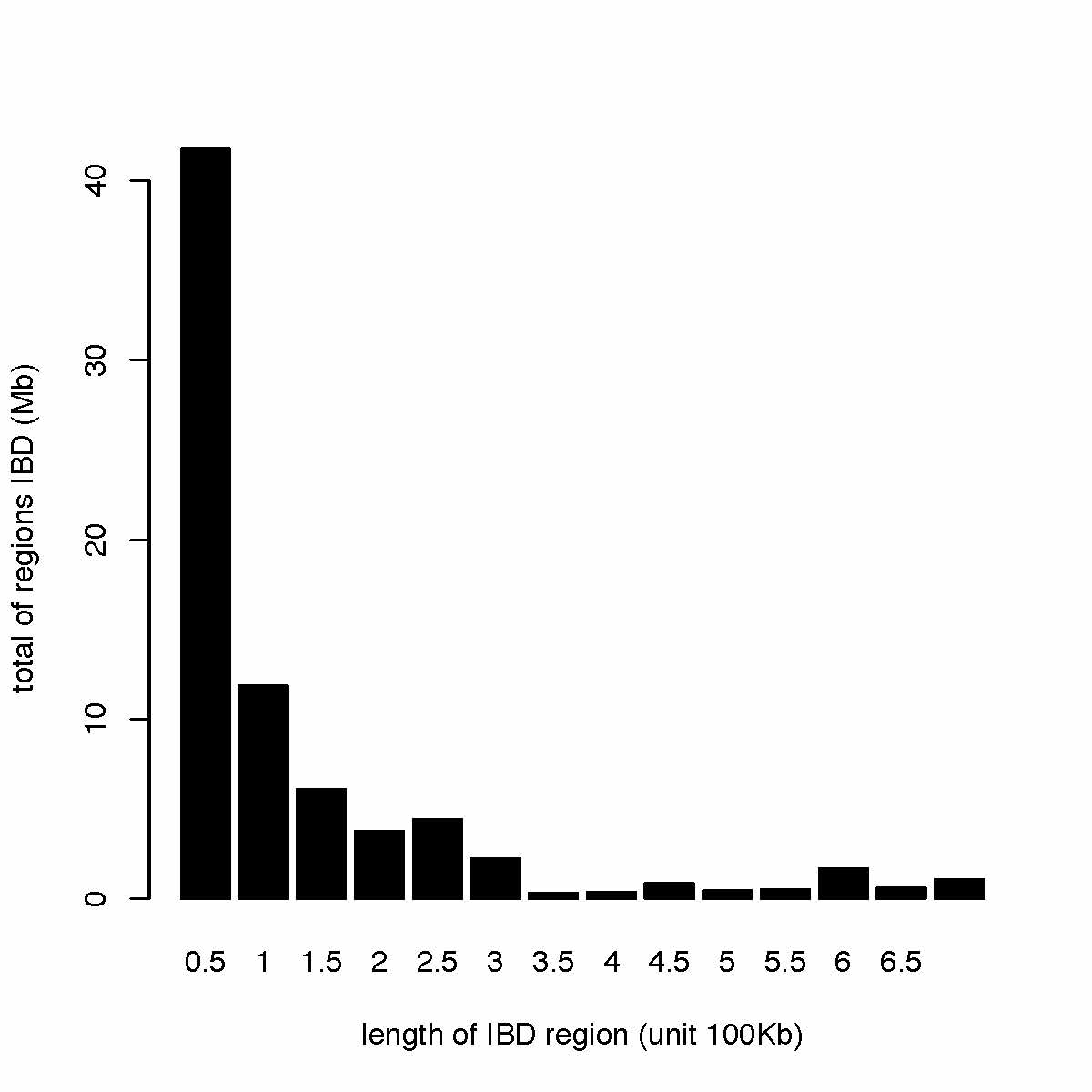


Supplementary Table 1. Collection site information.

| Latitude | Longitude | **Name** |
| --- | --- | --- |
| 49.7428 | -74.7359 | CHA001 |
| 49.7211 | -74.7616 | CHA003 |
| 49.6851 | -74.7743 | CHA004 |
| 49.5228 | -74.7292 | CHA005 |
| 49.6025 | -75.1591 | CHA007 |
| 50.5561 | -74.5983 | RDN002 |
| 50.5107 | -74.4891 | RDN003 |
| 50.5561 | -74.5983 | RDN004 |
| 50.5561 | -74.5983 | RDN005 |
| 45.28 | -81.72 | Fathom five |
| 44.84833 | -79.86143 | GBI |
| 43.53 | -80.31 | Guelph |
| 45.4 | -79.01 | Limberlost |
| 43.83 | -79.18 | Rouge |
| 43.71 | -79.96 | Silvercreek |
| 45.49 | -78.25 | Whitney |
| 44.82 | -79.98 | Awenda |
| 45.15 | -81.58 | Dorcas Bay |

Supplementary Table 2 – Gene Ontology categories for gene with low D and Pi

This is supplied as Excel file Supp_Table2_LowD_LowPi.xlsx.

Supplementary Table 3 – Candidate Immune Genes

Supplied as file “Candidate_ApisImmuneGenes_Supp.xls”. This gene set includes genes from a recent study on honey bee immune response(Ryabov et al., 2014) and bee orthologs of members of Gene Ontology Biological Process term "Immune System Process", GO:0002376, taken from Flybase.

Supplementary Table 4 – SnpEff results for genes in Table 2.

Supplied as Excel file “Supp_Table4_Table1SnpEff.xls”. This table contains results for each gene in Table 2, in the same order, describing the numbers and types of different mutations in the neighborhood of the gene.

References

Albrechtsen, A., Moltke, I., & Nielsen, R. (2010). Natural Selection and the Distribution of Identity-by-Descent in the Human Genome. *Genetics, 186*(1), 295-308. doi:10.1534/genetics.110.113977

Ryabov, E. V., Wood, G. R., Fannon, J. M., Moore, J. D., Bull, J. C., Chandler, D., . . . Evans, D. J. (2014). A virulent strain of deformed wing virus (DWV) of honeybees (Apis mellifera) prevails after Varroa destructor-mediated, or in vitro, transmission. *PLoS Pathogens, 10*(6), e1004230.
